# Influence of Interpersonal and Institutional Trust on the Participation Willingness of Farmers in E-Commerce Poverty Alleviation

**DOI:** 10.3389/fpsyg.2021.727644

**Published:** 2021-10-29

**Authors:** Guoyi Chen, Wei Tan, Shangmin Zhang, Bangquan Yan

**Affiliations:** ^1^Department of Business Management, Chongqing Three Gorges University, Chongqing, China; ^2^Department of Public Administration, Chongqing Three Gorges University, Chongqing, China

**Keywords:** interpersonal trust, institutional trust, e-commerce poverty alleviation, social trust, participation willingness

## Abstract

To explore the influence of interpersonal trust and institutional trust on the participation willingness of farmers in e-commerce poverty alleviation in China, a questionnaire survey of 320 farmers in Chongqing Ecological Tourism District was adopted for data collection, and a binary logistic model was used for data analysis. The results showed that (1) both interpersonal trust and institutional trust had a positive influence on the participation behavior of farmers in e-commerce poverty alleviation, and the priority ranking from high to low was: trust in government, trust in relatives, trust in neighbors, and trust in village cadres. (2) Institutional trust had a greater impact on the participation behavior of farmers than interpersonal trust, especially in the poverty-stricken areas where economic development was relatively backward. (3) Individual attributes, household attributes, and rural resource attributes had a significant positive impact on the participation intention of farmers. Among these, the role of rural e-business service platform was particularly important. The role of institutional trust at the village level still did not perform well in promoting the participation willingness of farmers. Based on empirical analysis, the suggestions for promoting the active cooperation of farmers and participating in the cooperation of e-business were put forward, such as enhancing the interpersonal network of farmers, improving the rural e-commerce information service platform, and strengthening the construction of the rural business environment.

## Introduction

Poverty eradication is a common mission of all mankind. Since 1978, China has achieved great success by lifting more than 600 million people out of poverty and the poverty incidence has dropped from 97.5% in 1978 to 1.7% in 2017 (He and Wang, 2019), which was called the fast large-scale poverty reduction in human history. However, according to the statistics of the Chinese National Rural Revitalization Bureau, by the end of 2019, there were 2,707 poor villages and 5.51 million people still lived in poverty in China (Liang et al., 2020). These people mainly lived in the mountain area of western China, most of which lack basic living conditions. Under the circumstances, the government of China, therefore, adjusted the direction of poverty alleviation and adopted the targeted e-commerce poverty alleviation strategy (Han et al., 2019).

Furthermore, with the rapid spread and growth of the Internet, the process of rural informatization is accelerating constantly. Rural e-commerce provides effective assistance for the implementation of targeted poverty reduction programs (Cui et al., 2017), and points out a new direction for promoting rural revitalization (Fosu, 2017). Particularly in recent years, the number of Taobao villages has increased from 20 in 2013 to 5,425 in 2020, which fully demonstrated the great potential of rural e-commerce poverty alleviation (Wang H. et al., 2020). Promoting E-commerce poverty alleviation plays an important role in accelerating the rural infrastructure construction, improving the marketization level of agricultural products as well as increasing the income of farmers (Han et al., 2019).

Therefore, this research aimed to explore and investigate the factors stimulating the participation of farmers in rural e-commerce poverty alleviation from a socioeconomic perspective. The existing studies have shown that social capital plays a significant role in the development of rural e-commerce poverty alleviation (Portes, 1998). While social capital covers multiple dimensions, huge differences exist between them (Kuang et al., 2019; Deng et al., 2020). Considering this, the present research chose “trust” as an independent variable, conducted a field survey, and finally analyzed the role of interpersonal trust and institutional trust in the participation of farmers in e-commerce poverty alleviation.

The previous research shows that there is a positive relationship between trust and cooperation (e.g., Lewicki and Bunker, 1996; Stern and Coleman, 2015; Zand, 2016). Lewicki and Bunker (1996) stated that trust is beneficial for reducing uncertainty and conflict among individuals. According to Luhmann (2000), trust is considered as the premise of cooperation and coordination interaction, and it can strengthen cooperation among individuals. In addition, when living in a high trust society, individuals will have a strong willingness for business, which can reduce the transaction costs and subsequently enhance the stability of cooperation (Wang and Wan Wart, 2007; Vries, 2014). Then, what is the role of social trust during the participation of farmers in e-commerce poverty alleviation, and what is the relationship between them? This study will address the issue by analyzing the cooperative participation behavior of farmers from the perspective of social trust, and reveal the internal relationship between the social trust and participating behavior of the farmers. It is expected to be beneficial for understanding the influencing factors of the participation of farmers in e-commerce poverty alleviation, enriching and improving academic literature in the related fields, and also providing a reference for the promotion of new rural construction.

The structure of this paper is organized as follows: Literature review and research hypothesis comprehensively reviewed the literature on the relationship between interpersonal and institutional trust and the cooperative participation of farmers in e-commerce operation, and put forward the research hypothesis, research methodology and data analysis described the data source, model selection, variables, and data processing. Results analysis analyzed the empirical research results of this paper and discussion, limitation, and recommendations presented a discussion of the findings and made a brief recommendation for the study. The last part concluded the whole research.

## Literature Review and Research Hypothesis

The establishment of interpersonal trust is based on family membership, blood relationships, neighborhood relations, etc., and it can be divided into two types, such as trust in relatives and trust in neighbors (Rus and Iglič, 2005; Lewicki et al., 2006). In recent years, scholars have made huge progress on the research of interpersonal trust and the e-commerce participation behavior of farmers (Kong et al., 2014). First, Feng et al. (2016) stated that interpersonal trust could reduce transaction costs and promote cooperation. In rural society, the interpersonal relationships among the individual farmers are of highly geographically characteristics, and information transmission is mainly realized through the non-institutional channels, such as village meetings (Feng et al., 2016), communication of neighbors (Hoell, 2004), and media networks (Evans and Revelle, 2008). Interpersonal trust can enhance the communication and sharing of information between the different social groups, and promote farmers to accept e-commerce (Sønderskov, 2011). Second, interpersonal trust has a positive influence on the e-commerce operation decisions of farmers (Wang et al., 2019). This mutual trust among the farmers can promote their communication and cooperation, which is beneficial for forming the mechanism of risk-sharing and benefit-sharing, and makes the cooperation more efficient (Six, 2007). During rural e-commerce business development, getting assistance from relatives and friends can significantly improve the production efficiency of farmers (Zeng and Xia, 2019). Based on this, this research proposes the following hypotheses:

Hypothesis 1: Interpersonal trust has a significant impact on the participative behavior of farmers in e-commerce operations. The higher the level of interpersonal trust, the stronger the participating enthusiasm of the farmer becomes.

According to Daskalopoulou (2019), the formation of institutional trust is based on the contract institutional environment, and institutional trust is established on the “non-interpersonal” relationship. Institutional trust affects the decision-making of the participation willingness of farmers through the internal constraint mechanism (Hudson, 2006). Moreover, it can usually be divided into two types, such as trust in the government and trust in village cadres (Hudson, 2006; Fuglsang and Jagd, 2015).

The past research has confirmed the role of institutional trust in improving the participation intention of farmers. Hudson (2006) found that the trust and reciprocity between the farmers and governmental departments are conducive to the market promotion of agricultural products in the European Union (EU). Chen et al. (2019) discussed the relationship between village social capital and the participation of rural households in e-commerce, and they found that general trust and institutional trust had a positive impact on the participation intention. Sharp and Smith (2003) found that the social trust mechanism has a positive impact on the willingness of individuals to participate in e-commerce. Based on the above, this paper proposes the following hypotheses:

Hypothesis 2: Institutional trust has a significant impact on the participative behavior of farmers in e-commerce operations. The higher the level of institutional trust is, the stronger the participating enthusiasm of farmers becomes.

Xin and Zhou (2012) proposed that under the diversity-orderly structure of Chinese native soil society, emotion can effectively promote the initial participation of farmers. However, as the interpersonal relationships became estranged, the role of the mechanism and the institutional system becomes more and more important (de Vries et al., 2019). At the same time, Domanski and Artur (2021) found that policy and institutional trust plays a much more important role than interpersonal trust in promoting the participation willingness of farmers, especially in the poverty-stricken region. Therefore, this paper proposes the following hypotheses:

Hypothesis 3: Institutional trust has a greater impact on the participation intention of farmers than interpersonal trust. Especially in the poverty-stricken region with an underdeveloped economy, the role of institutional trust is particularly important.

As to the study of the participation behavior of farmers, Lastra-Bravo et al. (2015) and Riley et al. (2018) reveal that the individual attributes of farmers, such as educational background, risk appetite, and perception of e-commerce project indeed have a relation with the participation behavior. Lee and Ihm (2020) stated that gender has a relation with the participation willingness of farmers, and they added that male farmers were more likely to participate in group activities while the female farmers preferred private activities. Another survey conducted by Shi et al. (2010) proposed that the participation willingness was a predictor of participation behavior, and it was also affected by gender. Men tended to have a stronger willingness to participate in rural business activities, for they were considered as the main contributor of family income (Wang et al., 2019). Besides, the surveys conducted by Song (2016) have shown that individual attributes, such as age and educational level, had an impact on their participation intention. And the level of education was found to be positively related to the participation willingness of farmers, the experience of by-business also affected their participation behavior (Zepeda, 2009). Kong et al. (2019) proposed that by-business farmers were less willing to participate in rural e-commerce actively for they had more ways to increase the income. Huang (2012) also added that the understanding of rural e-commerce was also a significant predictor of the participation intention of farmers. Above all, the previous studies discussed individual dimensions just from one or two attributes, lack of comprehensive and systematic review. Thus, this research integrates four attributes together, such as gender, age, education level, and perception of e-commerce. We thus propose the following hypotheses:

Hypothesis 4a: Individual attributes have a significant positive impact on the participation intention of farmers, and with the increase of the proportion of male, age, educational level, and understanding of e-commerce poverty alleviation, the participation willingness of farmers becomes stronger.

The studies had shown that the participation willingness was also influenced by family agricultural acreage, annual household income, and the number of the labor force, and there was a significant positive relation among them (Liu and Song, 2002; Liu and Lai, 2016). The larger the family agricultural acreage was, the easier it was to realize economies of scale, and the more willing the farmer was to participate in e-commerce business (Hu, 2010). Similarly, Smithers et al. (2008) found that the number of labor forces had a positive relation with the family participation willingness. In general, the families with a high proportion of e-commerce income depended more on e-commerce poverty alleviation, spent more time on learning e-commerce technologies, and were more likely to participate in e-commerce poverty alleviation (Hou and Ding, 2016). Based on the above, we thus propose the following hypotheses:

Hypothesis 4b: Household attributes have a significant positive impact on the participation intention of farmers, and with the increase of family agricultural acreage, annual household income, and the number of the labor force, the participation willingness of farmers becomes stronger.

The previous studies mostly focus on these endogenous resources, while neglecting the influence of external rural resources (Wang J. et al., 2020). The distance to the market (Menapace et al., 2016), road accessibility (Liu, 2013), allocation of village officials (Lienhoop and Brouwer, 2015), and the situation of the e-business service station (Christensen et al., 2011; Jin et al., 2015), all these factors have posed an influence on the participation intention of farmers. Accessibility is the foundation for developing rural commercial circulation (Wu and Zhang, 2012), such as both road accessibility and informational service accessibility, especially the latter one. Incomplete and missing information would reduce the enthusiasm of farmers for the online transaction (Van Weele et al., 2016). Therefore, the timelier information farmers attained, the smoother their transaction was, and the greater the likelihood of participation was. Besides, as the leader of rural grassroots organizations, college-graduate village officials owned advanced information technology and market information, they were also proved to be positively related to the participation intention of farmers (Morris et al., 2017). Thus this paper put forward the following hypotheses:

Hypothesis 4c: Rural resource attributes have a significant positive impact on the participation intention of farmers.

**Figure 1:**
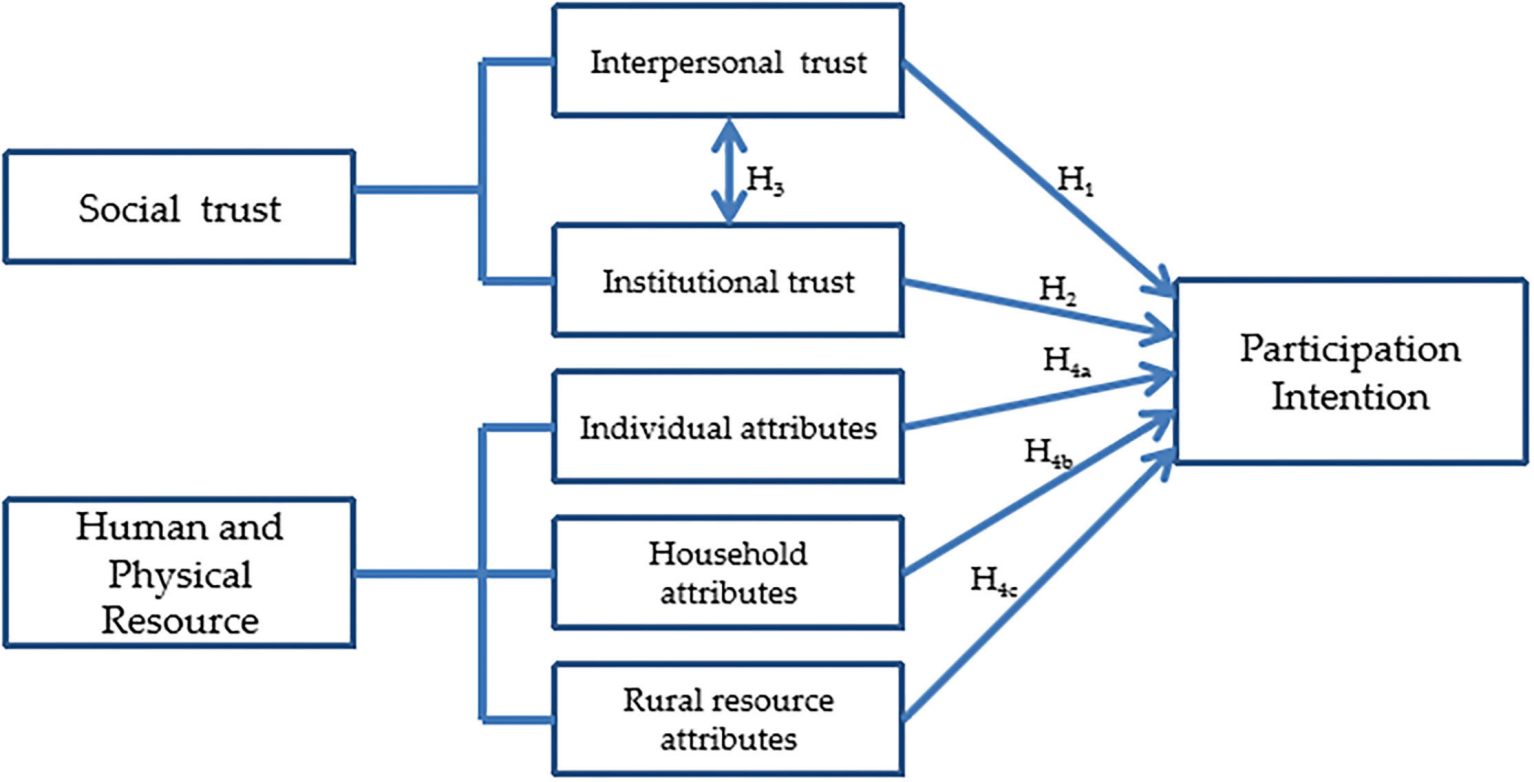
Theoretical framework.

In summary, the social trust and human and physical resource dimension may have an influence on the participation intention of farmers. Figure 1 describes the relationship among them and presents the research framework of this study.

## Research Methodology and Data Analysis

### Research Sample and Method

Wanzhou District is located in the northeast of Chongqing, China (Zou et al., 2018), with abundant agricultural resources and advantaged eco-tourism conditions, making it suited for developing featured e-commerce agricultural products (Peng et al., 2017). A series of electric commodity brands, such as Yuquan pickled cabbage, Master Ran beef jerky, and Wanzhou Guhong orange, are well-known in China [Zhang and Deng, 2010; CQS (Chongqing Statistics), 2019]. Given this, to examine whether the social trust has significantly affected the participation behavior of farmers, the research group conducted an on-spot survey of 26 villages in Wanzhou from July 16 to August 25, 2020. In the survey, 320 farmers participated of which 295 valid questionnaires were collected.

The sample had the following characteristics: the majority of households were male, accounting for 73.2%; the average age of the respondents was 53.4 years old, the youngest was 28 years, and the oldest was 79 years old. Their education level is mainly junior high school, accounting for 43.4%, followed by the primary school or below, accounting for 40%, and the rest are of senior high school or above degree, accounting for 16.6%. The respondents were all married.

### Research Modeling

A discrete choice model was derived from the study of animal binary reflection conditions by Fecher in 1860 (Hall et al., 2002). In the 1970s and 1980s, this model was widely used in the research of economic decision-making, such as economic layout, firm location, traffic problem, employment problem, and purchase decision (Hall et al., 2002; de Bekker-Grob et al., 2013). This study mainly discusses the influence of social trust on the participation behavior of farmers. Therefore, the dependent variable of this study is “behavior of farmer,” that is “whether to participate in e-business or not,” which belongs to a 0–1 variable. According to the outcomes of model fitting, the binary logistic model based on the individual level was selected to analyze the key factors affecting the behavior of farmers. As to the participation behavior of farmers in e-commerce poverty alleviation, the following equation is:


$$\begin{array}{l} \log it(Behavior,=1)=\phi (\alpha_{i}trust_{i}+\beta_{i}individul_{i}+\gamma_{i}household_{i}\\ \;+\delta_{i}village_{i} \end{array}$$

In the formula, the subscript *i* represent the farmer been surveyed. Behavior is a dependable variable ranging from 0 to 1, which described the cooperative participation of farmers in e-commerce operations. If farmers cooperate in e-commerce operation, its value is 1; otherwise, it is 0.

### Variables and Data Processing

#### Dependent Variable: Participation Behavior of Farmers

The dependent variable of this study is “whether farmers participate in e-commerce poverty alleviation.” Among the 295 valid samples obtained, 55 participated in e-commerce, accounting for 18.64%, and 240 were non-participants, accounting for 81.36%. Therefore, the cooperative participation of farmers in e-commerce operations is relatively low. Table 1 shows the results of the cooperative participation of farmers in e-commerce operations. It is found that the participation rate of male farmers is higher than that of female ones. Similarly, the participation rate of highly educated farmers is higher than that of low-educated ones. In contrast, the participation willingness of high-income farmers is weaker than that of low-income ones.

**Table 1:** The results of the participating respondents in e-commerce poverty alleviation.

**Index**	**All farmers**	**Sex**		**Household income**		**Education**	
		**Male**	**Female**	**Highincome**	**Low income**	**Highlyeducated**	**Lowly educated**
Participation	18.64	21.3	15.19	16.27	21.36	49.49	11.19
Non-participation	81.36	78.7	84.81	83.73	78.64	50.51	88.81

#### Independent Variable: Trust

Trust was an independent variable in this study. According to the measurement of trust variables in the study of Bitmiş and Ergeneli (2013) and Asveld et al. (2015), this research chose two variables to measure interpersonal trust, they trust in relatives (trust_1_) and trust in neighbors (trust_2_), and the two variables to measure institutional trust, they trust in village cadres (trust_3_) and trust in the government (trust_4_). The respondents were asked to fill in the blanks with the Likert scale, namely, “strongly disagree,” “disagree,” “generally,” “agree,” and “strongly agree,” which were assigned 1–5 points, respectively. The size and significance of α_i_ are the core of this study. If trust can indeed enhance the participation behavior of farmers, the significance of α_i_ is positive. Table 2 presented the result of the influence of social trust on the participation of farmers. It can be seen from Table 2 that trust_1_, trust_2_, trust_3_, and trust_4_ have the same trend. That is to say, the higher the degree of trust was, the higher the participation intention of farmers was.

**Table 2:** Influence of trust on the participation behavior of farmers.

	**Index**	**Items**	**Participation**	**Non-participation**	**Total**
Interpersonal trust	Trust_1_	Not at all	0	100	100
		No trust	4.15	95.85	100
		Normal	5.63	94.37	100
		Trust	18.76	81.24	100
		Fully trust	50.17	49.83	100
	Trust_2_	Not at all	0	100	100
		No trust	0	100	100
		Normal	6.73	93.27	100
		Trust	21.72	78.28	100
		Fully trust	41.18	58.82	100
Institutional trust	Trust_3_	Not at all	0	100	100
		No trust	2.36	97.64	100
		Normal	13.74	86.26	100
		Trust	37.82	62.18	100
		Fully trust	63.76	36.24	100
	Trust_4_	Not at all	0	100	100
		No trust	1.15	98.85	100
		Normal	6.42	93.52	100
		Trust	33.52	66.48	100
		Fully trust	67.12	32.88	100

#### Control Variable

To explore the role of trust in the participation of farmers in e-commerce operations, the construction of control variables is indispensable. Based on the previous research, this study constructed five variables related to the individual attributes of the farmers, three variables about household attributes, and three variables of rural resource attributes, and finally formed the required control variables system. The specific variables involved in this study and their processing methods are shown in Table 3.

**Table 3:** The descriptive statistics of variables.

**Variable**	**Definition and assignment**		**Mean**	**Std. Err**.
**Dependent Variable**				
—-Participate in e-commerce	Yes = 1,no = 0		0.186	0.373
business nor not			
**Independent Variable**				
— Trust_1_	1 = Not at all, 2 = no trust, 3 = normal,4 = trust, and 5 = fully trust		3.710	0.857
— Trust_2_	1 = Not at all, 2 = no trust, 3 = normal,4 = trust, and 5 = fully trust		3.609	0.867
— Trust_3_	1 = Not at all, 2 = no trust, 3 = normal,4 = trust, and 5 = fully trust		2.969	0.971
— Trust_4_	1 = Not at all, 2 = no trust, 3 = normal,4 = trust, and 5 = fully trust		3.212	1.012
**Control Variable**				
Individual attributes	sex	1 means male and 0 means female	0.715	0.459
	age	1 means 30 ~ 39, 2 = 40 ~ 49,3 = 50 ~ 59,4 = 60 ~ 69, 5 = 70 ~ 79	2.811	0.511
	education	1 = primary school,2 = junior, 3 = high school,4 = college or above	1.856	0.827
	by-business	1 = yes 2 = No	0.621	0.493
	Cognition of rural e-commerce	1 = Not at all, 2 = no, 3 = normal,4 = understand, and 5 = fully understand	2.711	0.974
Household attributes	Family agricultural acreage	The size of family agricultural acreage	0.389	0.416
	Labor force amount	the number of labor force 5 = 70 ~ 79	2.953	1.183
	Household income	Annual household income	3.532	1.783
Rural resource attributes	Road accessibility	1 = worst, 2 = worse, 3 = normal,4 = better, and 5 = best	3.516	1.899
	Rural information service station	1 = yes 0 = No	0.278	0.478
	College student village officer	1 = yes 0 = No	0.296	0.431

## Results Analysis

The SPSS 22.0 statistical software (IBM, NY, USA) was used in this study for empirical analysis. Since the dependent variable is 0–1, this study adopts a binary logistics regression model to fit and analyse the factors affecting the participation behavior of farmers. Moreover, the second-order regression was chosen to study the influence of social trust, which can describe the outcomes clearly. As to the research procedure, first, all the control variables were introduced into the regression equation to test the significance of the regression coefficients (Johnson and Onwuegbuzie, 2004), and Model 1 was obtained. Then, on this basis, four variables of social trust were introduced into the regression equation (Georgakopoulos et al., 2020), and the significance test of the regression coefficients of all variables was carried out to obtain Model 2. Finally, the overall explanatory power gap between the two models was compared and analyzed in Table 4.

**Table 4:** The regression results of different models.

**Variable**		**Model 1**			**Model 2**		
		**B**	**S.E**.	**Exp(B)**	**B**	**S.E**.	**Exp(B)**
Trust	Trust_1_				0.913**	0.395	2.536
	Trust_2_				0.797**	0.390	2.242
	Trust_3_				0.723**	0.312	2.101
	Trust_4_				1.285***	0.457	3.763
Individual attributes	sex	1.243*	0.745	3.456	1.413***	0.567	4.558
	Age	−0.04	0.398	0.912	−0.017	0.276	0.983
	Education	1.136***	0.399	3.141	1.006***	0.298	3.012
	By-business	−0.986***	0.254	1.956	−0.913***	0.313	2.012
	Perception of rural E-business	0.187***	0.291	1.216	0.615	0.217	1.728
Household attributes	Family agricultural acreage	1.075***	0.359	2.929	1.139***	0.251	3.123
	Labor force amount	−2.098*	1.165	0.142	−2.003**	0.867	0.135
	Household income	−0.419***	0.191	0.658	−0.611***	0.156	0.543
Rural resource attributes	Road accessibility	0.32***	0.301	1.410	0.311***	0.221	1.359
	Rural E-business service platform	1.326**	0.651	3.776	1.505***	0.451	4.643
	College student village officer	0.72	0.847	2.038	1.415	0.515	4.351
Constant		−18.718	3.659	0	−6.508	1.801	0.001
Chi-Square		107.159			169.559		
-2 log likelihood		171.033			108.632		
Nagelkerke R^2^		0.492			0.724		
% Correct prediction rate		89.600			93.2		

*, **, and *** respectively indicated significant at 10, 5, and 1% levels.

The regression results showed that both Model 1 and Model 2 had passed the test at the significance level of 1%, and Nagelkerke *R*^2^ of each model reached 49.2 and 72.4%, respectively, and the correct prediction rate reached 89.6 and 93.2%, respectively. After adding the dimensions of social trust, Nagelkerke R^2^ of Model 2 increased by 20.8% and the correct prediction rate also increased by 3.6%. These data showed that social trust indeed enhanced the Nagelkerke R^2^ of each variable. The regression results of Model 2 indicated that trust_1_, trust_2_, trust_3_, and trust_4_ all have a significant correlation with the participation willingness of the farmers. It can be seen that both interpersonal trust and institutional trust play a very important role in the decision-making of participation of farmers.

Specifically, trust in relatives (trust_1_) passed the test at the significance level of 5%. That is, with other conditions unchanged, the probability of the participation willingness of farmers will be 2.536 times than before, while trust_1_ doubles. The reason underlying this is that frequent visits between relatives can communicate and share information and reduce the cost of searching for cooperative partners (Sønderskov, 2011; Van Oortmerssen et al., 2014). At the same time, under the mutual trust of relatives, the cooperative contract is easy to be reached and tends to be stable, which can also effectively avoid opportunistic behaviors (Sønderskov, 2011). Therefore, trust in relatives has a significant positive influence on the e-commerce participation willingness of farmers.

Trust in neighbors (trust_2_) passed the test at the significance level of 5%. That is, with other conditions unchanged, the probability of the participation willingness of farmers will be 2.242 times than before while trust_2_ doubles. The explanation may be that frequent interactions of rural villagers lead to form an atmosphere of mutual understanding (Ajenaghughrure et al., 2020). According to Özcan and Bjørnskov (2011), it not only increased the mutual sense of identity but also reduced the transaction costs of business cooperation, thus strengthened their desire to cooperate. With the continuous improvement of the trust of farmers in their neighbors, their cooperation willingness gradually becomes clear, and finally, a cooperation mechanism of risk-sharing and benefit-reciprocity is also formed (Kwak et al., 2019).

The above two sets of data indicate that interpersonal trust (the trust of relatives and neighbors) has a significant impact on the decision-making of participation of farmers. The higher interpersonal trust is, the higher the participation willingness of farmers will be. So H_1_ is supported.

The trust in village cadres (trust_3_) passed the test at the significance level of 5%. That is, with other conditions unchanged, the probability of the participation willingness of farmers will be 2.101 times than before while trust_3_ doubles. A possible explanation may be that with the constant improvement of the trust of farmers in village cadres, the rural community gradually formed a kind of trust relations (Li et al., 2019), this can reduce the risk and uncertainty, and enhance the confidence of farmers through policy support and technical guidance from village cadres. The existing studies have confirmed that as agents of grass-roots political power (Tang and Zhu, 2020), the village governing organizations communicated and implemented national policies during e-commerce poverty alleviation (Tang and Zhu, 2020; Wang H. et al., 2020), and had posed influence on the e-business implementation practice.

The trust in government (trust_4_) passed the test at the significance level of 1%. That is, with other conditions unchanged, the probability of the participation willingness of farmers will be 3.763 times than before while trust_4_ doubled. Li et al. (2019) proved that trust in government has a positive influence on the participation willingness of farmers.

The above two sets of data indicate that institutional trust (trust in village cadres and trust in the government) has a significant influence on the behavioral decisions of farmers' participation. The higher the level of institutional trust becomes, the higher the participation willingness of farmers will become. H_2_ is supported.

In addition, among the four dimensions of trust, trust_3_ has the lowest score, indicating that village cadres and governance have not played a great role in the development of rural trust. The underlying reason was that the rural governance had undergone significant changes in the post-agricultural tax era, then the carrier of state power in the countryside became weakened (Tatarko, 2014), and the connection between the farmers and the village-level government was getting more and more looser (Lumineau et al., 2015). This kind of flat organizational structure (Seppänen et al., 2007) prompted farmers to directly communicate with the national policy and state regulations, thus weakened the discretionary power of village cadres. Especially in recent years, with the implementation of favorable policies, such as “new rural cooperative medical care” (Li et al., 2019) and “direct grain subsidy” (Hu et al., 2019), which directly connected the farmers with the central government, thus the influence of village cadres became weaker and weaker. Thus, H_3_ is true.

The influence of other control variables on the participation willingness of farmers is analyzed below.

1. From the perspective of individual attributes of farmers: both the variables, such as gender and education level, all have passed the significance test at the confidence level of 1%, and both coefficients were positive, indicating that male farmers are more likely to participate in e-commerce operation in comparison with the female farmers. The higher level of education of the farmers increased the possibility to participate. In addition, the variable of by-business has a significant influence on the participation willingness of farmers; the number of farmers without by-business is 21.52% higher than those with by-business. The underlying reasons are that farmers without multiple occupations depend more on local development (Lastra-Bravo et al., 2015; Hu et al., 2019), and they actively participate for increasing income. In addition, the perception of rural e-commerce of farmers also passed the test at the significance level of 5%, that is, the higher understanding of rural e-commerce farmers have, the greater possibility of participation will become. However, the variable of age did not pass the significance test. So, hypothesis H_4a_ is partially supported.
2. From the perspective of household attributes: family agricultural acreage and family annual income pass the significance test at the confidence level of 1%, and the regression coefficient of family agricultural acreage is positive, indicating the family that has more arable land area is more likely to participate in e-commerce cooperation. The coefficient of annual family income is negative, indicating that low-income farmers are more inclined to participate in e-commerce operations than high-income farmers. In addition, the labor force passes the test at the 5% significance level. So, hypothesis H_4B_ is partially supported.
3. From the perspective of rural resource attributes: rural e-business service platform, traffic accessibility, and college student village officer, all pass the test at the significance level of 1%, and the coefficient is positive, which indicate that they all have a significant positive influence on the participation willingness of farmers. So H_4c_ is supported.

It is worth noting that, after adding the factors of social trust, the variable of “village officer” and “perception of rural e-commerce” were proved to have no significant correlation with the participation willingness of farmers. The Nagelkerke R_2_ of the overall model increased by 20.7%. This indicates that social trust plays a greater role in the decision-making of the participation of farmers than other factors.

Table 5 shows the regression results of the two groups, the low-income group and the low-education group. The results indicated that (1) farmers of the low-income group are affected by trust_1_, trust_2_, and trust_3_. (2) Farmers of the low-education group were affected by trust_3_ and trust_4_, and neither trust_1_ nor trust_2_ has any influence on their participation willingness. It can be concluded that social trust can increase the likelihood of participation of farmers, and institutional trust has a greater impact on the decision-making of farmers than interpersonal trust. Moreover, the population of the poverty-stricken region mainly consisted of low-education and low-income ones. Therefore, it can be inferred that institutional trust plays a particularly important role in the poverty-stricken region of an underdeveloped economy. So, hypothesis H_3_ is also supported.

**Table 5:** The cluster regression results.

**Variable**	**Low-income**		**Lowly-educated**	
	**B**	**Exp(B)**	**B**	**Exp(B)**
Trust_1_	1.585** (0.474)	4.706	0.902 (0.587)	2.548
Trust_2_	1.386*** (0.533)	4.051	0.601 (0.489)	1.783
Trust_3_	1.083*** (0.497)	2.962	1.065* (0.445)	2.843
Trust_4_	0.889 (0.565)	1.993	1.298** (0.574)	3.686
Control variable	YES		YES	
Chi-square	133.531		87.789	
−2 log likelihood	74.815		67.963	
Nagelkerke R^2^	0.737		0.622	

*, **, and *** respectively indicated significant at 1, 5, and 10% levels.

## Discussion, Limitation, and Recommendations

### Discussion

What remains to be answered is how social trust contributes to the participation willingness of farmers in e-commerce poverty alleviation. Therefore, in this study, we attempted to reveal the underlying mechanism of the influence of interpersonal trust and institutional trust on the participation willingness of farmers in e-commerce poverty alleviation in China. Based on the questionnaire survey of 295 farmers in Chongqing eco-tourism district, the results showed that (a) both interpersonal trust and institutional trust all had a significant influence on the participation of farmers in e-commerce poverty alleviation, (b) compared with interpersonal trust, the institutional trust had a greater impact on the participation intention of farmers in China, and (c) rural resource dimensions all had a significant positive influence on the participation willingness of farmers, while individual attributes and household attributes did not pass the significance test.

First, this study explored whether social trust, such as institutional trust and interpersonal trust promoted the participation intention of farmers. The result revealed the positive influence of both institutional and interpersonal trust on the participation intention. This result is consistent with the previous research conducted by Sønderskov (2011) and Wang et al. (2019). These findings stated that kinship between the relatives and frequent interactions between rural neighbors increased the mutual sense of identity and reduced the cost of searching for cooperative partners. In addition, the governments gradually performed well in policy communication and information service (Chen et al., 2019), some local governments had recruited college-graduate village officials for providing e-commerce technical support, all these increased the confidence of farmers in e-commerce poverty alleviation, thus, it can be concluded that the higher the level of social trust is, the stronger the participating enthusiasm of farmers becomes.

Second, the study compared the influence of institutional and interpersonal trust and revealed that institutional trust has a greater impact on the participation intention of farmers. This finding is in line with the previous research, which stated that the institutional trust had a significant effect on the participation willingness of farmers for its increasingly influence on the rural governance structure and normative expectations of individual farmers (Fuglsang and Jagd, 2015; Chen et al., 2019). Especially in the remote poverty-stricken areas with scarce resources, most people were low-education and low-income ones, the farmers depended more on the government to obtain business opportunities and preferential policies. Therefore, the role of institutional trust was much more important in the mind of farmers. In addition, from the perspective of Hofstede's national cultural dimension, China belongs to a society of high collectivism and huge power distance (Gelfand et al., 2006). Gelfand et al. (2011) had supplemented that the individuals in tight societies when compared with the loose ones, tended to rely more on the institutional norms with clear expectations enforced through national authority. Therefore, it is not surprising in China that the rural farmers depended more on national institutions and state governments for resource-seeking and problem-solving. Thus, compared with interpersonal trust, institutional trust had a greater impact on the participation intention of farmers.

Third, this study integrated human and physical resource dimensions into this theoretical framework and explored their influence on the participation intention of farmers. The results showed that (a) with the increase of the proportion of male farmers, educational level, by-business, and understanding of e-commerce poverty alleviation, the participation willingness of farmers became stronger. The research found that the participation rate of highly educated was 38.3% higher than the lower ones. This can be explained as following: the operation of e-business requires farmers to master the basic knowledge of internet technology and software applications (Xu, 2016), and a higher level of education and understanding enhances their online service competence, thus increasing their willingness to participate. Besides in a traditional agricultural society, men assumed more financial responsibility for the family (Huang, 2012), and thus, they were more likely to adopt e-commerce for family income than women. The farmers of by-business had more ways for increasing income (Kong et al., 2019), their participation rate is relatively low than the ones with no other choice. (b) The dimensions of household attributes had a significant positive impact on the participation intention of farmers, with the increase of family agricultural acreage and the number of the labor force, the participation willingness of farmers became stronger. A possible explanation was that both family labor force and land resources were important resources for e-commerce production (Liu and Lai, 2016). These resources enhance their confidence and willingness to participate. Besides, the research found that the participation rate of high family income was 38.3% lower than that of low ones. A possible reason was that the high family-income families may have more ways for increasing their income, thus, their participation willingness was lower. (c) Rural resource dimensions, such as rural e-business service platform, traffic accessibility, and College student village officer, all had a significant positive influence on the participation willingness of farmers. Among these, the role of rural e-business service platforms was particularly important. The underlying reason may be that it is an informational infrastructure for business development. It not only brought advanced technology and operation experience but also an opened-up huge market for the poor rural areas (Hou and Ding, 2016), thus greatly enhanced the participation willingness of the farmers.

Last, the result of the presented study highlighted that the institutional trust at the village level did not perform well in promoting the participation willingness of farmers. Among all the four independent variables, trust in village cadres (trust_3_) is the weakest. It is, therefore, necessary and urgent to improve the administrative service skills of rural grassroots organizations in the future.

### Limitation and Policy Implications

There are many limitations that may be addressed by further research. First, 320 farmers in Chongqing province were selected for investigation; the representativeness of the research population is limited. The scope of the sample should be expanded to another agricultural province of China. Second, under the influence of COVID-19, the frequency of face-to-face communication and interviews was reduced, thus, a questionnaire survey was adopted for data collection. Insufficient data could lead to result bias. Further research could integrate the questionnaire survey and experimental design with archival data to enrich the data source. Third, this study mainly focused on the influence of social trust on the participation willingness of farmers in e-commerce poverty alleviation, however, the mediating paths of attitude and subjective norms should also be investigated in the future. In addition, other influencing factors, such as human capital and physical capital should be investigated in the future study.

Based on the above, the following implications were made for encouraging the participation of farmers: (1) interpersonal trust: on the one hand, the rural government should initiatively carry out series of activities to strengthen the interpersonal relationship network of farmers, such as establishing the rural community organizations and holding informational exchange meetings regularly. On the other hand, rural wealth-leader or opinion leaders should be selected and trained. The rural government should formulate a policy to identify, train, and develop these talents with leadership competence, entrepreneurial spirit, and relevant professional skills. The aim is to stimulate them to play a leading and exemplary role in e-commerce participation and operation. (2) Institutional trust: it is necessary to improve the disclosure system of rural government information about e-business operation, and it is of great urgency to establish some non-governmental organizations to lead and supervise the behavior of village cadres (Han and Yan, 2019). At the same time, the farmers should be encouraged to actively participate in the village public affairs, and keep frequently in contact with the village cadres, thus their trust in the local government and village cadres will be enhanced. (3) Rural physical resource: strengthen the construction of rural infrastructure and create a good condition for rural e-business. First, the rural infrastructure construction, such as rural logistics, wireless networks, and rural information service stations should be strengthened. Second, it is necessary to transform the traditional household small-scale workshop into large-scale mass production for reducing the production cost as well as improving the product quality. Finally, the construction of a rural public security environment should be strengthened, thus the willingness of farmers to participate in e-commerce operations will be enhanced.

## Conclusion

The study emphasized the role of social trust in promoting the participation willingness of farmers in e-commerce poverty alleviation. The findings showed that both institutional and interpersonal trust had a significant positive impact on the participation intention of farmers. The higher the level of social trust was, the stronger the participation willingness of farmers becomes. In addition, compared with interpersonal trust, institutional trust has a greater impact on the participation intention of farmers. Especially in poverty-stricken areas where economic development is relatively backward, the role of institutional trust is particularly important. Furthermore, the role of institutional trust at the village level still did not perform well in promoting the participation willingness of farmers. It is urgent and necessary to take measures to improve the trust of farmers in village cadres. It is expected that these findings will enrich the existing literature of social trust, enhance the participation enthusiasm of farmers, thus promoting the development of rural e-commerce.

## Data Availability Statement

The original contributions presented in the study are included in the article/supplementary material, further inquiries can be directed to the corresponding author.

## Ethics Statement

Ethical review and approval was not required for the study on human participants in accordance with the local legislation and institutional requirements. The patients/participants provided their written informed consent to participate in this study.

## Author Contributions

GC and SZ: conceptualization and validation. GC: methodology and project administration. WT: software, investigation, and visualization. BY: formal analysis, resources, supervision, and funding acquisition. SZ: data curation and writing—review and editing. GC: writing—original draft preparation. All authors have read and agreed to the published version of the manuscript.

## Funding

This study was funded by the Humanities and Social Sciences Research Program of Chongqing Municipal Education Commission (Grant No. 17SKG163), the Science and Technology Research Program of Chongqing Municipal Education Commission (Nos. KJQN202101213, KJQN201901243, and KJQN202001240), and the Teaching Reform Projects of Chongqing Three Gorges University (No. JGYB1803).

## Conflict of Interest

The authors declare that the research was conducted in the absence of any commercial or financial relationships that could be construed as a potential conflict of interest.

## Publisher's Note

All claims expressed in this article are solely those of the authors and do not necessarily represent those of their affiliated organizations, or those of the publisher, the editors and the reviewers. Any product that may be evaluated in this article, or claim that may be made by its manufacturer, is not guaranteed or endorsed by the publisher.

## References

[B1] Ajenaghughrure I. B. Sousa S. D. C. Lamas D. (2020). Measuring trust with psychophysiological signals: a systematic mapping study of approaches used. Multimodal Technol. Interact. 4:63. 10.3390/mti4030063

[B2] Asveld L. Ganzevles J. Osseweijer P. (2015). Trustworthiness and responsible research and innovation: the case of the bio-economy. J. Agric. Environ. Ethics 28, 571–588. 10.1007/s10806-015-9542-2

[B3] Bitmiş M. G. Ergeneli A. (2013). The role of psychological capital and trust in individual performance and job satisfaction relationship: a test of multiple mediation model. Procedia Soc. Behav. Sci. 99, 173–179. 10.1016/j.sbspro.2013.10.483

[B4] Chen L. A. Miranda B. V. Parcell J. L.. (2019). The foundations of institutional-based trust in farmers' markets. Agric. Hum. Values. 36, 395–410. 10.1007/s10460-019-09923-4

[B5] Christensen T. Pedersen A. B. Nielsen H. O. Mørkbak M. R. Hasler B. Denver S. (2011). Determinants of farmers' willingness to participate in subsidy schemes for pesticide-free buffer zones—a choice experiment study. Ecol. Econ. 70, 1558–1564 10.1016/j.ecolecon.2011.03.021

[B6] CQS (Chongqing Statistics) (2019). Statistical Communiqué of Chongqing on 2019 National Economic and Social Development. Available online at: http://data.tjj.cq.gov.cn/easyquery.htm?cn=D0101 (accessed February 12, 2021).

[B7] Cui M. Pan S. L. Newell S. Cui L. (2017). Tactics, resource orchestration and e-commerce enabled social innovation in Rural China. J Strategic Inf. Syst. 26, 3–21. 10.1016/j.jsis.2016.10.001

[B8] Daskalopoulou I. (2019). Individual-level evidence on the causal relationship between social trust and institutional Trust. Soc. Indic. Res. 144, 275–298. 10.1007/s11205-018-2035-8

[B9] de Bekker-Grob E. W. Rose J. M. Bliemer M. C. J. (2013). A closer look at decision and analyst error by including nonlinearities in discrete choice models: implications on willingness-to-pay estimates derived from discrete choice data in healthcare. Pharmaco Econ. 31, 1169–1183. 10.1007/s40273-013-0100-3 24178372

[B10] de Vries J. R. van der Zee E. Beunen R. Kat R. Feindt P. H. (2019). Trusting the people and the system. The interrelation between interpersonal and institutional trust in collective action for agri-environmental management. Sustainability 11:7022. 10.3390/su11247022

[B11] Deng X. Zeng M. Xu D. Qi Y. (2020). Does social capital help to reduce farmland abandonment? Evidence from big survey data in rural china. Land 9:360. 10.3390/land9100360

[B12] Domanski H. Artur P. (2021). The relation between interpersonal and institutional trust in European countries: which came first? Pol. Sociol. Rev. 213, 87–102. 10.26412/psr213.05

[B13] Evans A. M. Revelle W. (2008). Survey and behavioral measurements of interpersonal trust. J. Res. Pers. 42, 1585–1593. 10.1016/j.jrp.2008.07.011

[B14] Feng Z. Vlachantoni A. Liu X. (2016). Social trust, interpersonal trust and self-rated health in China: a multi-level study. Int. J. Equity Health 15:180. 10.1186/s12939-016-0469-7 27825358 PMC5101682

[B15] Fosu A. K. (2017). Growth, inequality, and poverty alleviation in developing countries: recent global evidence. Res Econ. 71, 306–336. 10.1016/j.rie.2016.05.005

[B16] Fuglsang L. Jagd S. (2015). Making sense of institutional trust in organizations: bridging institutional context and trust. Organization 22, 23–39. 10.1177/1350508413496577

[B17] Gelfand M. J. Nishii L. H. Raver J. L. (2006). On the nature and importance of cultural tightness-looseness. J. Appl. Psychol. 91, 1225–1244. 10.1037/0021-9010.91.6.1225 17100480

[B18] Gelfand M. J. Raver J. L. Nishii L. Leslie L. M. Lun J. Lim B. C.. (2011). Differences between tight and loose cultures: A 33-nation study. Science. 332:1100–1104. 10.1126/science.1197754 21617077

[B19] Georgakopoulos I. Chalikias M. Zakopoulos V. Kossieri E. (2020). Identifying factors of students' failure in blended courses by analyzing students' engagement data. Educ. Sci. 10:242. 10.3390/educsci10090242

[B20] Hall J. Kenny P. King M. Louviere J. Viney R. Yeoh A. (2002). Using stated preference discrete choice modelling to evaluate the introduction of varicella vaccination. Health Econ. 11, 457–465. 10.1002/hec.694 12112494

[B21] Han G. Yan S. (2019). Does food safety risk perception affect the public's trust in their government? An empirical study on a national survey in China. Int. J. Environ. Res. Public Health 16:1874. 10.3390/ijerph16111874 31141881 PMC6603658

[B22] Han J. Wang J. Ma X. (2019). Effects of farmers' participation in inclusive fnance on their vulnerability to poverty: evidence from Qinba poverty-stricken area in China. Emerg. Mark. Financ. Tr. 55, 998–1013. 10.1080/1540496X.2018.1523789

[B23] He H. Wang S. (2019). A review of rural pro-poor tourism effect in China from a multidimensional perspective. Chin. J. Agric. Resour. Reg. Plann. 40, 180–187. 10.7621/cjarrp.1005-9121.20190423

[B24] Hoell R. C. (2004). The effect of interpersonal trust and participativeness on union member commitment. J. Bus. Psychol. 19, 161–177. 10.1007/s10869-004-0546-6

[B25] Hou J. Ding Z. (2016). An empirical study on the performance evaluation of entrepreneurship policy for peasant workers in jiangxi province. World Surv. Res. 19–22.

[B26] Hu J. (2010). Analysis of the realistic factors constraining farmers to return home to start businesses-survey from Jintang county of Sichuan province of China. Rural Econ. 113–116.

[B27] Hu Q. Xu Q. Xu B. (2019). Introducing of online channel and management strategy for green agri-food supply chain based on pick-your-own operations. Int. J. Environ. Res. Public Health 16:1990. 10.3390/ijerph16111990 31167493 PMC6603932

[B28] Huang G. (2012). Measurement scale of residents' perception, participant capacity and willingness on the poverty elimination effect by tourism. J. Anhui Agric. Sci. 40, 3439–3441. 10.3969/j.issn.0517-6611.2012.06.085

[B29] Hudson J. (2006). Institutional trust and subjective well-being across the EU. Kyklos 59, 43–62. 10.1111/j.1467-6435.2006.00319.x

[B30] Jin J. Gao Y. Wang X. Nam P. K. (2015). Farmers' risk preferences and their climate change adaptation strategies in the Yongqiao District, China. Land Use Policy 47, 365–372. 10.1016/j.landusepol.2015.04.028

[B31] Johnson R. B. Onwuegbuzie A. J. (2004). Mixed methods research: a research paradigm whose time has come. Educ. Res. 33, 14–26. 10.3102/0013189X033007014

[B32] Kong D. T. Dirks K. T. Ferrin D. L. (2014). Interpersonal trust within negotiations: meta-analytic evidence, critical contingencies, and directions for future research. Acad. Mange. J. 57, 1235–1255. 10.5465/amj.2012.0461

[B33] Kong F-Z. Zhao L. Zhang X-B. Tsai C-H. Lin D. D. (2019). Farmers' Work-Life Quality and Entrepreneurship Will in China. Front. Psychol. 10:787. 10.3389/fpsyg.2019.00787 31114517 PMC6502897

[B34] Kuang F. Jin J. He R. Wan X. Ning J. (2019). Influence of livelihood capital on adaptation strategies: evidence from rural households in Wushen Banner, China. Land Use Policy 89:104228. 10.1016/j.landusepol.2019.104228

[B35] Kwak J. Zhang Y. Yu J. (2019). Legitimacy building and e-commerce platform development in China: the experience of Alibaba. Technol. Forecast. Soc. Change 139, 115–124. 10.1016/j.techfore.2018.06.038 32287407 PMC7127782

[B36] Lastra-Bravo X. B. Hubbard C. Garrod G. Tolón-Becerra A. (2015). What drives farmers' participation in euagri-environmental schemes?: results from a qualitative meta-analysis. Environ. Sci. Policy. 54, 1–9. 10.1016/j.envsci.2015.06.002

[B37] Lee J. Ihm J. (2020). Gender difference in returns to education independent of gender wage gap in Korea. Asian. Econ. J. 34, 213–232. 10.1111/asej.12209

[B38] Lewicki R. Bunker B. (1996). Developing and maintaining trust in work relationships, in Trust in Organizations, eds Kramer R. Tyler T. (Thousand Oaks, CA: Sage), 114–139. 10.4135/9781452243610.n7

[B39] Lewicki R. J. Tomlinson E. C. Gillespie N. (2006). Models of interpersonal trust development: theoretical approaches, empirical evidence, and future directions. J. Manag. 32, 991–1022. 10.1177/0149206306294405

[B40] Li L. Du K. Zhang W. Mao J. Y. (2019). Poverty alleviation through government-led e-commerce development in rural China: an activity theory perspective. Inf. Syst. J. 29, 914–952. 10.1111/isj.12199

[B41] Liang X. Wei H. Xia Q. Yu J. (2020). The efficiency and path of poverty alleviation by tourism in my country's concentrated contiguous destitute areas. Stat. Decis. 36, 16–20. 10.13546/j.cnki.tjyjc.2020.21.003

[B42] Lienhoop N. Brouwer R. (2015). Agri-environmental policy valuation: farmers' contract design preferences for afforestation schemes. Land Use Policy 42, 568–577. 10.1016/j.landusepol.2014.09.017

[B43] Liu E. M. (2013). Time to change what to sow: risk preferences and technology adoption decisions of cotton farmers in China. Rev. Econ. Stat. 95, 1386–1403. 10.1162/REST_a_00295

[B44] Liu G. Song H. (2002). Farmers returning home to start businesses: characteristics, driving forces, and its influence-case analysis of 71 farmers returning home to start businesses. China. Rural.Econ. 65–71.

[B45] Liu Y. Lai X. (2016). Literature review of farmers returning home to start businesses. Rural Econ. Sci. Technol. 207–209.

[B46] Luhmann N. (2000). Familiarity, confidence, trust: problems and alternatives, in Trust: Making and Breaking Cooperative Relations, ed Gambetta D. (Oxford: University of Oxford).

[B47] Lumineau F. Guo S. L. Lewicki R. J. (2015). Revisiting the foundations of organizational distrust. Soc. Sci. Electron. Publ. 1, 1–88. 10.1561/3400000001

[B48] Menapace L. Colson G. Raffaelli R. (2016). A comparison of hypothetical risk attitude elicitation instruments for explaining farmer crop insurance purchases. Eur. Rev. Agric. Econ. 43, 113–135. 10.1093/erae/jbv013

[B49] Morris M. H. Shirokova G. Tsukanova T. (2017). Student entrepreneurship and the university ecosystem: a multi-countryn empirical exploration. Eur. J. Int. Manage. 11, 65–85. 10.1504/EJIM.2017.081251

[B50] Özcan B. Bjørnskov C. (2011). Social trust and human development. J. Socio Econ. 40, 753–762. 10.1016/j.socec.2011.08.007

[B51] Peng Y. Zhou F. Cui J. Du K. Leng Q. Yang L.. (2017). Impact of socioeconomic and meteorological factors on reservoirs' air quality: a case in the Three Gorges Reservoir of Chongqing (TGRC), China over a 10-year period. Environ. Sci. Pollut. Res. 24, 16206–16219. 10.1007/s11356-017-9221-0 28540543

[B52] Portes A. (1998). Social capital: its origins and applications in modern sociology. Annu. Rev. Sociol. 24, 1–24. 10.1146/annurev.soc.24.1.1

[B53] Riley M. Sangster H. Smith H. Chiverrell R. Boyle J. (2018). Will farmers work together for conservation? The potential limits of farmers' cooperation in agri-environment measures. Land Use Policy 70, 635–646. 10.1016/j.landusepol.2017.10.049

[B54] Rus A. Iglič H. (2005). Trust, governance and performance: the role of institutional and interpersonal trust in sme development. Int. Sociol. 20, 371–391. 10.1177/0268580905055481

[B55] Seppänen R. Blomqvist K. Sundqvist S. (2007). Measuring inter-organizational Trust—A critical review of the empirical research in 1990–2003. Ind. Mark. Manag. 36, 249–265. 10.1016/j.indmarman.2005.09.003

[B56] Sharp J. Smith M. (2003). Social capital and farming at the rural-urban interface: the importance of nonfarmer and farmer relations. Agric. Syst. 76, 913–927. 10.1016/S0883-2927(02)00083-5

[B57] Shi Z. Tan Y. Wu H. (2010). Analysis of entrepreneurship behaviors and entrepreneurship will of farmers returning home. China Rural Surv. 25–27.

[B58] Six F. E. (2007). Building interpersonal trust within organizations: a relational signaling perspective. J. Manag. Gov. 11, 285–309. 10.1007/s10997-007-9030-9

[B59] Smithers J. Lamarch J. Joseph A. (2008). Unpacking the terms of engagement with local food at the farmers' market: insights from Ontario. J. Rural Stud. 24, 337–350. 10.1016/j.jrurstud.2007.12.009

[B60] Sønderskov M. K. (2011). Explaining large-N cooperation: generalized social trust and the social exchange heuristic. Ration. Soc. 23, 51–74. 10.1177/1043463110396058

[B61] Song Y. (2016). Institutionalizing rural women's political participation in China: reserved seats election for women. Asian Women 32, 77–99. 10.14431/aw.2016.09.32.3.77

[B62] Stern M. J. Coleman K. J. (2015). The multidimensionality of trust: applications in collaborative natural resource management. Soc. Nat. Resour. 28, 117–132. 10.1080/08941920.2014.945062

[B63] Tang W. C. Zhu J. (2020). Informality and rural industry: rethinking the impacts of e-commerce on rural development in china. J. Rural Stud. 75, 20–29. 10.1016/j.jrurstud.2020.02.010

[B64] Tatarko A. (2014). Trust, cooperative behavior and economic success: when trust is the capital of the person?, in Higher School of Economics Research Paper No. WP BRP 22/PSY/2014 (Moscow). 10.2139/ssrn.2521374

[B65] Van Oortmerssen L. A. van Woerkum C. M. J. Aarts N. (2014). The visibility of trust: exploring the connection between trust and interaction in a dutch collaborative governance boardroom. Public Manag. Rev. 16, 666–685. 10.1080/14719037.2012.743578

[B66] Van Weele M. V. Rijnsoever F. J. V. Nauta F. (2016). You can't always get what you want: how entrepreneur's perceived resource needs affect the incubator's assertiveness. Technovation 59, 18–33. 10.1016/j.technovation.2016.08.004

[B67] Vries J. R. D. (2014). Understanding Trust: Longitudinal Studies on Trust Dynamics in Governance Interactions. Wageningen: Wageningen University.

[B68] Wang H. Wen T. Han J. (2020). Can government financial inflows effectively reduce poverty in poverty-stricken areas? Evidence from China. Emerg. Mark. Financ. Tr. 56, 2461–2473. 10.1080/1540496X.2019.1618264

[B69] Wang J. Yang C. Ma W. Tang J. (2020). Risk preference, trust, and willingness-to-accept subsidies for pro-environmental production: an investigation of hog farmers in China. Environ. Econ. Policy Stud. 22, 405–431. 10.1007/s10018-020-00262-x

[B70] Wang S. Li Y. Tu Y. (2019). Linking proactive personality to life satisfaction in the Chinese context: the mediation of interpersonal trust and moderation of positive reciprocity beliefs. J. Happiness Stud. 20, 2471–2488. 10.1007/s10902-018-0056-2

[B71] Wang X. Wan Wart M. (2007). When public participation in administration leads to trust: an empirical assessment of managers' perceptions. Public Adm. Rev. 67, 265–278. 10.1111/j.1540-6210.2007.00712.x

[B72] Wu L. Zhang F. T. (2012). The entrepreneurial choice of rural migrant workers in countryside:from the perspective of entrepreneurial environment dimensionality. China. Popul. Res. Environ. 22, 116–120.

[B73] Xin Z. Q. Zhou Z. (2012). A cross-temporal meta-analysis of changes in Chinese college students' interpersonal trust. Adv. Psychol. Sci. 20, 344–353. 10.3724/SP.J.1042.2012.00344

[B74] Xu X. (2016). Research on factors influencing young farmers' employment quality. World. Surv. Res. 13–18. 10.1016/j.jstrokecerebrovasdis.2015.04.040 26215135

[B75] Zand D. E. (2016). Reflections on trust and trust research: then and now. J. Trust Res. 6, 63–73. 10.1080/21515581.2015.1134332

[B76] Zeng Y. Xia L. X. (2019). The relationship between interpersonal responsibility and interpersonal trust: a longitudinal study. Curr. Psychol. 38, 1182–1189. 10.1007/s12144-018-0083-5

[B77] Zepeda L. (2009). Which little piggy goes to market? Characteristics of US farmers' market shoppers. Int. J. Consum. Stud. 33, 250–257. 10.1111/j.1470-6431.2009.00771.x

[B78] Zhang J. F. Deng W. (2010). Industrial structure change and its eco-environmental influence science the establishment of municipality in Chongqing, China. Procedia Environ. Sci. 2, 517–526 10.1016/j.proenv.2010.10.056

[B79] Zou X. Li Y. Wei Z. Wang T. Hu Y. Zhu Y.. (2018). Population data and forensic efficiency of 21 autosomal STR loci included in AGCU EX22 amplification system in the Wanzhou Han population. Int. J. Legal Med. 132, 153–155. 10.1007/s00414-017-1680-9 28866789

